# A predictive analytics approach to improve telecom's customer retention

**DOI:** 10.3389/frai.2025.1600357

**Published:** 2025-08-29

**Authors:** Asem Omari, Omaia Al-Omari, Tariq Al-Omari, Suliman Mohamed Fati

**Affiliations:** ^1^Computer Information Systems, Higher Colleges of Technology, Al Ain, United Arab Emirates; ^2^Information Systems Department, College of Computer and Information Sciences, Prince Sultan University, Riyadh, Saudi Arabia; ^3^Department of Computer Science, Jordan University of Science and Technology, Irbid, Jordan

**Keywords:** customer retention, prediction, SVM, logistic regression, KNN, Naive Bayes

## Abstract

Customer retention is a critical challenge for telecom companies, and understanding customer churn can significantly improve business strategies. This paper focuses on developing an accurate predictive model to identify potential customer churn using advanced data analysis techniques. By applying machine learning algorithms, our aim is to improve decision-making processes and enable telecom providers to take proactive measures to retain customers. Through this research, we seek to gain deeper insight into customer behavior, ultimately helping telecom companies improve service offerings and reduce churn rates. We developed and evaluated a diverse set of predictive models using a dataset representing customer churn. Our comparative analysis highlights the strengths and weaknesses of various techniques, and among the developed models, the Support Vector Machine (SVM) achieved the highest performance. The main contribution of this study lies in integrating effective data pre-processing, feature selection, and interpretability into churn prediction models, thus addressing the gaps identified in earlier research.

## 1 Introduction

Customer churn prediction is a vital challenge in the telecommunications industry, as retaining existing customers is more cost-effective than acquiring new ones. Accurate prediction models enable telecom companies to implement proactive strategies that reduce churn rates, improve customer satisfaction, and improve business performance. Previous research has explored various machine learning models such as logistic regression, decision trees, random forests, and deep learning approaches like ChurnNet (Saha L. et al., 2023), which showed promising accuracy levels. However, many of these models either struggle to handle imbalanced datasets, lack explainability, or require extensive computational resources. This research aims to leverage data analysis and predictive modeling techniques to identify key factors influencing customer turnover and develop an effective predictive model. In our research approach, we focus on critical steps such as data pre-processing, feature selection, and model development to ensure accurate and actionable insights. Unlike prior works that often overlook interpretability or ethical implications, our study integrates transparency and responsible data handling practices throughout the modeling process. Ethical considerations, including data privacy and regulatory compliance, are also prioritized to maintain responsible data practices. By making predictive models interpretable, our aim is to provide telecom companies with actionable insights to improve customer retention strategies.

This study will explore various stages of data processing and modeling, including data preprocessing through handling missing values, outliers, and preparing the dataset for analysis. Then, Exploratory Data Analysis (EDA) is applied to help understand feature distributions, variable relationships, and churn patterns. Feature selection is also used to identify the most relevant attributes for customer churn prediction. After that, we evaluated different predictive modeling techniques to determine the most effective approach taking into account ethical considerations by ensuring compliance with data privacy regulations and maintaining responsible use of customer data. We evaluated several machine learning algorithms: Support Vector Machines (SVM), Logistic Regression, KNN, and Naive Bayes, and then compared their performance using real-world churn data. This allows us to build on previous efforts while addressing limitations in feature handling and model reliability.

Through this research paper, our objective is to demonstrate the power of data-driven decision making in solving real-world business challenges while ensuring transparency and ethical responsibility. The findings will contribute to the broader field of customer analytics and help telecom companies implement more effective retention strategies. Our contribution lies in presenting a balanced framework that prioritizes both predictive power and interpretability, offering telecom providers practical tools for improving customer engagement and reducing customer churn. A comprehensive methodology followed in our study, structured in five stages: data acquisition, pre-processing and transformation, feature selection, predictive model development, and evaluation. This paper is structured as follows: In Section 2 we discuss some previous and related work. Then, in Section 3, we explore the dataset, understand the attributes, handle missing data and outliers, study the skewness of the data, and perform the required transformation to make the dataset ready to be analyzed. After that, in Section 4, we build our predictive models and discuss our findings. Finally, in Section 5 we summarize and discuss future work.

## 2 Literature review

Customer churn prediction remains a critical focus in the telecommunications industry, as retaining existing customers is more cost-effective than acquiring new ones. Accurate prediction models enable companies to implement proactive strategies, thereby reducing churn rates and enhancing customer satisfaction. This literature review examines the evolution of predictive modeling techniques and their applications in churn prediction, incorporating recent advancements and methodologies.

Early approaches to churn prediction relied on statistical methods logistic regression and decision trees which helped in understanding churn behavior. In Mozer et al. (2000) utilized artificial neural networks to predict subscriber dissatisfaction, demonstrating the potential of machine learning in churn prediction. The advent of advanced computational methods led to a shift toward machine learning algorithms. Support vector machines (SVMs), random forests, and ensemble methods became prevalent due to their ability to handle complex, high-dimensional data. Vafeiadis et al. (2015) compared various machine learning techniques, concluding that ensemble methods often outperform single classifiers in predicting churn. However, these traditional models often lack interpretability and struggle with highly imbalanced datasets. More recently, Saha S. et al. (2023) introduced a deep learning approach, ChurnNet, which enhanced the prediction accuracy in the telecommunication sector. Although ChurnNet achieves high performance, it requires significant computational resources and remains a “black-box” model, limiting interpretability.

Effective churn prediction relies heavily on the quality of data and the features used. Data mining techniques have been employed to extract relevant patterns from large datasets. Kaur (2017) emphasized the importance of data pre-processing and feature selection in enhancing model accuracy. Similarly, Abdullaev et al. (2023) leveraged meta-heuristics with artificial intelligence to improve feature selection processes, leading to more accurate churn predictions. Recognizing that customer behavior is influenced by social interactions, researchers have incorporated social network analytics into churn prediction models. Óskarsdóttir et al. (2020) demonstrated that integrating social network features improves the predictive performance of churn models. Zhou et al. (2023) further advanced this approach by employing ensemble learning techniques to analyze social networks, resulting in early and accurate churn detection.

Deep learning approaches have also been explored for their ability to model complex, non-linear relationships in data. Fujo et al. (2022) applied deep learning techniques to churn prediction, achieving notable improvements in accuracy over traditional methods. Building upon this, Saha S. et al. (2023) developed a deep churn prediction method tailored for the telecommunication industry, which outperformed existing models in various performance metrics. However, as predictive models become more sophisticated, ethical considerations regarding data usage have come to the forefront.

Ensuring customer privacy and adhering to data protection regulations are paramount. Studies have called for transparent modeling practices and the implementation of robust data governance frameworks. For instance, Wu et al. (2021) proposed an integrated churn prediction and customer segmentation framework that emphasizes ethical data handling and customer privacy. Despite advancements, challenges persist, including handling imbalanced datasets, ensuring model interpretability, and integrating real-time data. Future research is directed toward developing more transparent models, leveraging real-time analytics, and addressing ethical concerns in data usage. Zdziebko et al. (2024) explored optimizing customer retention using fuzzy-based churn modeling, addressing the challenge of imbalanced datasets and providing more interpretable results.

Several studies have shed light on the potential role of artificial intelligence, predictive analytics, and deep learning across different fields. Such studies provide information of considerable value that can also be applied in regard to the prediction of customer churn with respect to the telecom industry. Al-Omari et al. (2025) dealt with governance and ethical challenges associated with the adoption of AI in higher education. They noted the following concerning data privacy, algorithmic fairness, the general resistance to AI-driven learning: the model argues that robust regulatory frameworks with AI deployment and require transparency and accountability. Observation, development of biased prediction due to biased models by misinterpretation of human behavior, and AROI strategies found wanting due to the above considerations-were highlighted and emphasized from the time of customer churn prediction.

Similar potential problems loom for customer churn prediction, where data-driven insights have to be handled with extreme care to ensure compliance with data protection regulations in conjunction with producing predictive models that genuinely assist in augmenting customer retention strategies. Semary et al. (2023) began exploring the effectiveness of transformer-based models, such as RoBERTa, combined with LSTM and CNN techniques, for sentiment analysis. Their results exhibited high accuracies in complex text classification tasks and actualized how deep learning could identify significant patterns from unstructured text data. Customer churn predictions in the telecom industry will gain invaluable insights into trends of customer dissatisfaction through analyzing customer feedback, reviews, and sentiment patterns and thus prepare the telecom companies for proactive measures in enhancing customer engagement.

Rehman et al. (2025) carried out a comparative evaluation of facial emotion recognition (FER) techniques, comparing them against the performance of deep learning models such as CNNs to traditional machine learning classifiers like SVM, KNN, and Random Forests. They generally found that deep learning models surpassed traditional methods in identifying complex patterns, particularly in handling unstructured and noisy data. These facts would have much significance to churn prediction, allowing telecoms to harness deep learning techniques to crunch huge volumes of customer data in order to be able to identify faint behavioral patterns that signify churn potential.

**Figure 1:**
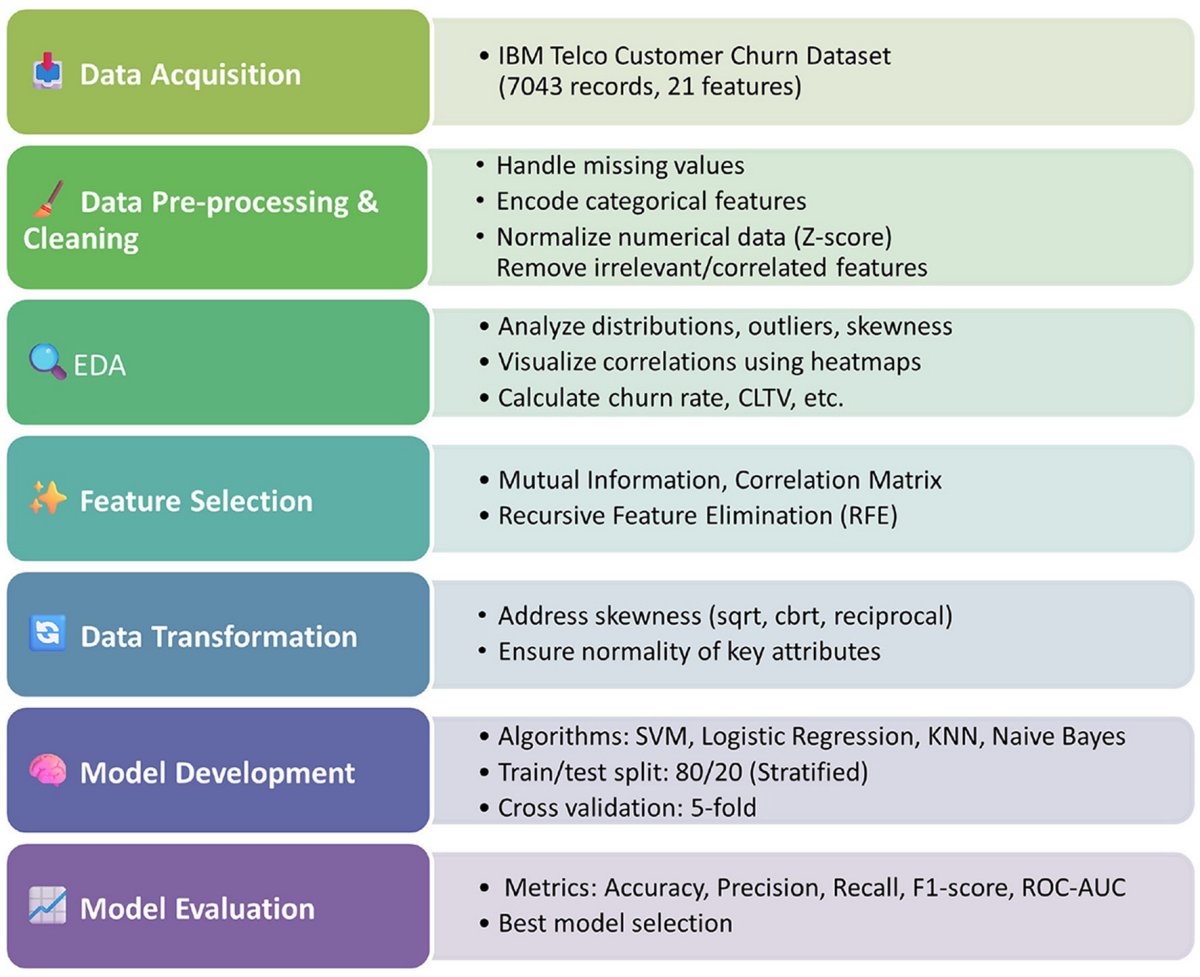
Methodology flowchart.

Jabbar et al. (2023) analyzed various text stemming techniques in NLP technologies and their benefits in enhancing text classifications and retrieval systems. Their research emphasized the strength of having good text pre-conditioning steps for improving classification performance. The cleansing of customer feedback, call transcripts, and textual data through these advanced NLP mechanisms could enhance model performance and improve prediction accuracy. In contrast to prior studies, our work emphasizes interpretable machine learning models with ethical design and preprocessing rigor, making it suitable for real-world telecom deployment. As the field continues to evolve, the integration of advanced machine learning, deep learning, and ethical data practices will play a crucial role in shaping the future of churn prediction in the telecommunications industry. In the following sections, we explain the full methodology used to develop, train, and evaluate the predictive models for customer churn analysis. Our methodology includes four phases: data preprocessing and cleaning, feature selection, model development, and model evaluation. Figure 1 shows the flowchart of our methodology starting from data acquisition all the way to model evaluation.

## 3 Data pre-processing and cleaning

We used the IBM Telco Customer Churn dataset from Kaggle (2024), a publicly available dataset widely used for churn prediction research. It contains 7043 customer records with 21 features (both numerical and categorical) related to demographic and service usage information. As a critical sector marked by intense competition and evolving customer preferences, understanding the factors influencing customer churn is paramount for sustainable business growth. This dataset has a diverse range of features that shed light on the ever-evolving dynamics of customer behavior within the tele-communications industry. We began our study with a detailed inspection of all columns to classify them into categorical, numerical, and binary features, which guides our cleaning and encoding processes. We performed multiple preprocessing steps to prepare the data for modeling: Invalid entries in “TotalCharges” were converted to NaN and imputed using the column mean, Categorical features were encoded using Label Encoding for binary columns (e.g., gender, Partner, Dependents). in addition to One-Hot Encoding for multiclass categorical columns (e.g., Contract, InternetService). Numeric features such as MonthlyCharges and Tenure were standardized using Z-score normalization. Outliers were visually inspected using boxplots and z-scores, with no extreme values requiring removal.

### 3.1 Feature selection

**Figure 2:**
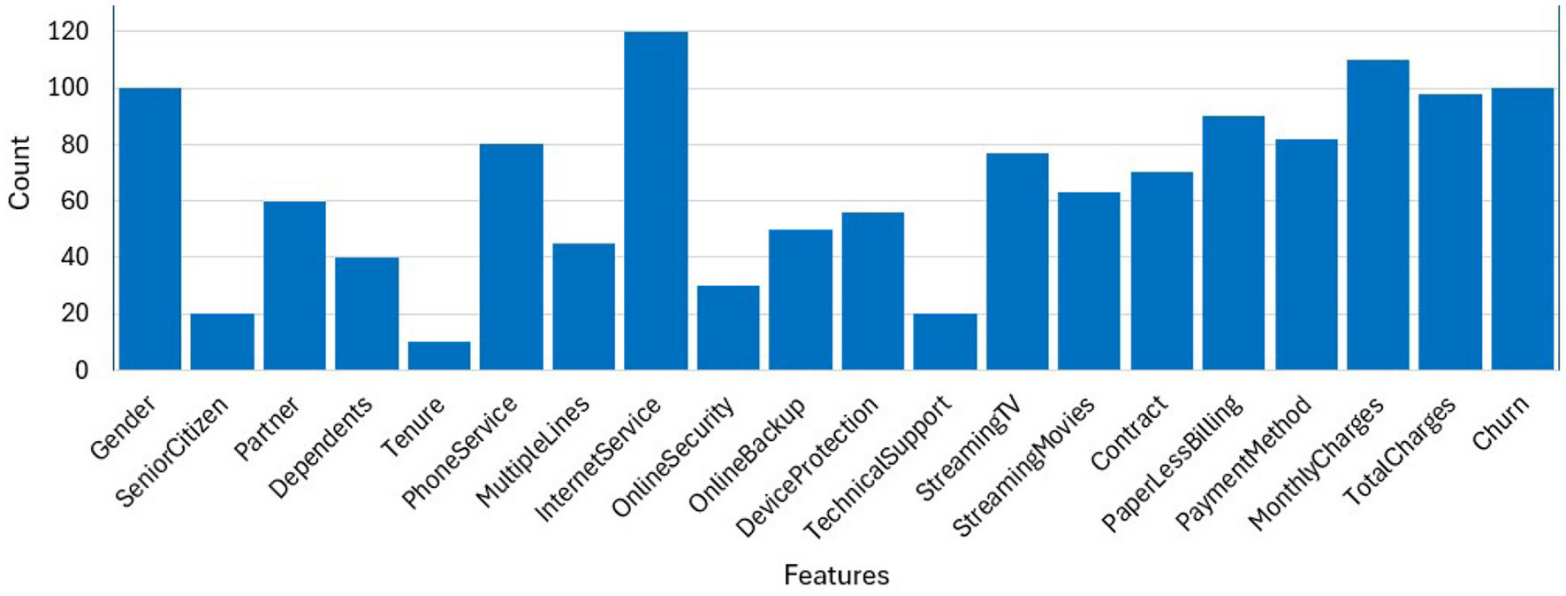
Frequency of dataset attributes.

Feature relevance was assessed using Correlation heatmaps (to detect multicollinearity), Mutual Information scores, and Recursive Feature Elimination (RFE) with logistic regression. We selected features such as Contract, Churn Score, Tenure, MonthlyCharges, and CLTV due to their demonstrated predictive power in our correlation analysis. The bar graph in Figure 2, interprets the count or frequency of each feature in the given dataset. Each bar represents a specific feature, and the height of the bar corresponds to the count or frequency of that feature within the dataset. We performed correlation analysis and mutual information ranking to identify features most relevant to churn.“Internet Service,” “Monthly Charges” and “Churn” occur the most, while “Tenure” and “Tech Support” occur the least number of times in the data set. Highly correlated features like “Zip Code” and “Latitude” were dropped.

**Figure 3:**
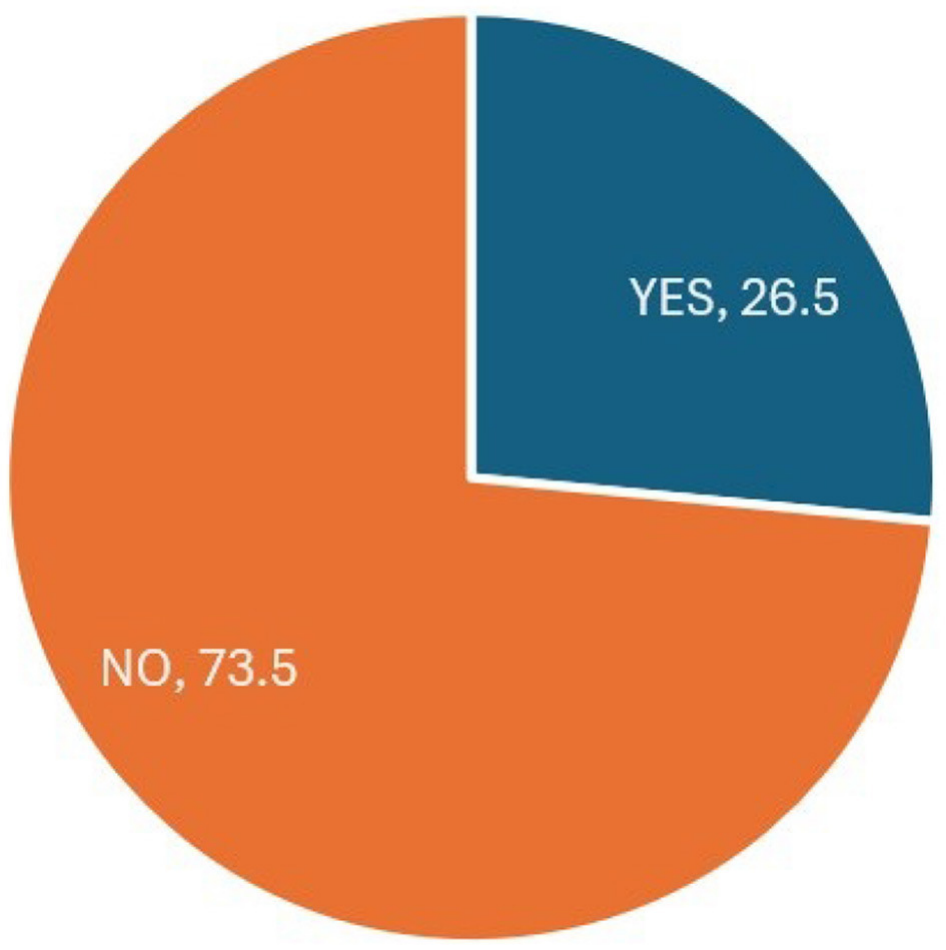
Distribution of the “Churn Label” variable.

Furthermore, the pie chart in Figure 3, illustrates the distribution of the “Churn Label” variable within the dataset. The chart reveals that a relatively smaller proportion, ~ 26.5%, represents customers who have chosen to discontinue their telecom services. In contrast, most customers, which make up around 73.5%, have opted to continue their services.

### 3.2 Exploratory analysis of the dataset

**Figure 4:**
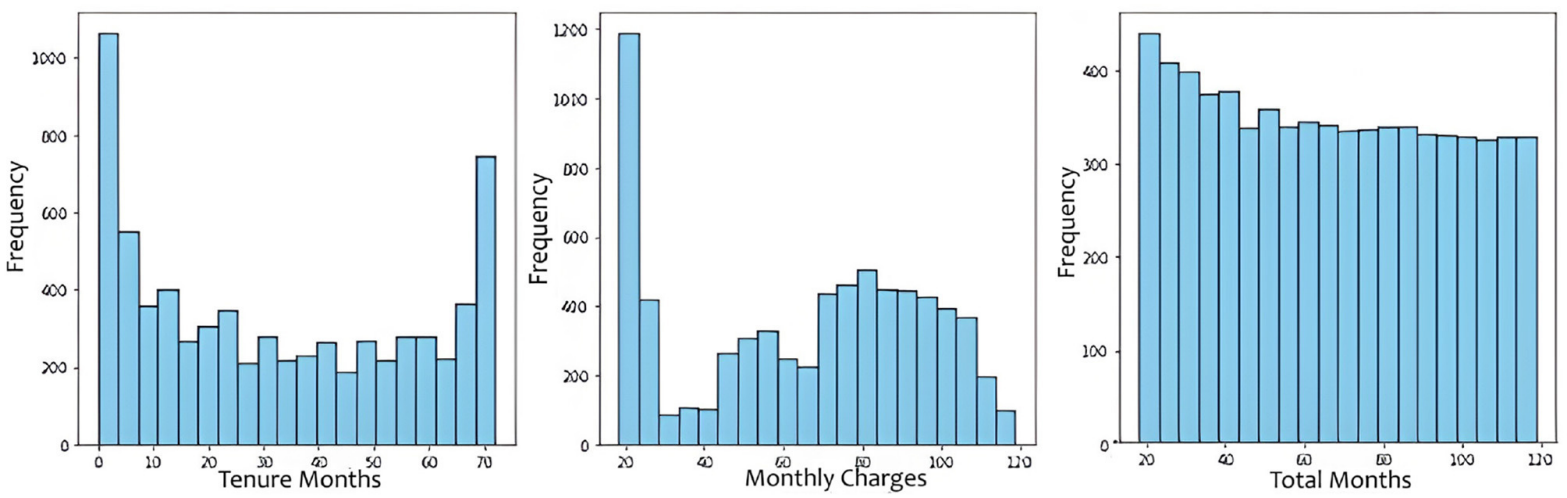
Frequency of tenure months, monthly charges and total charges in the dataset.

**Figure 5:**
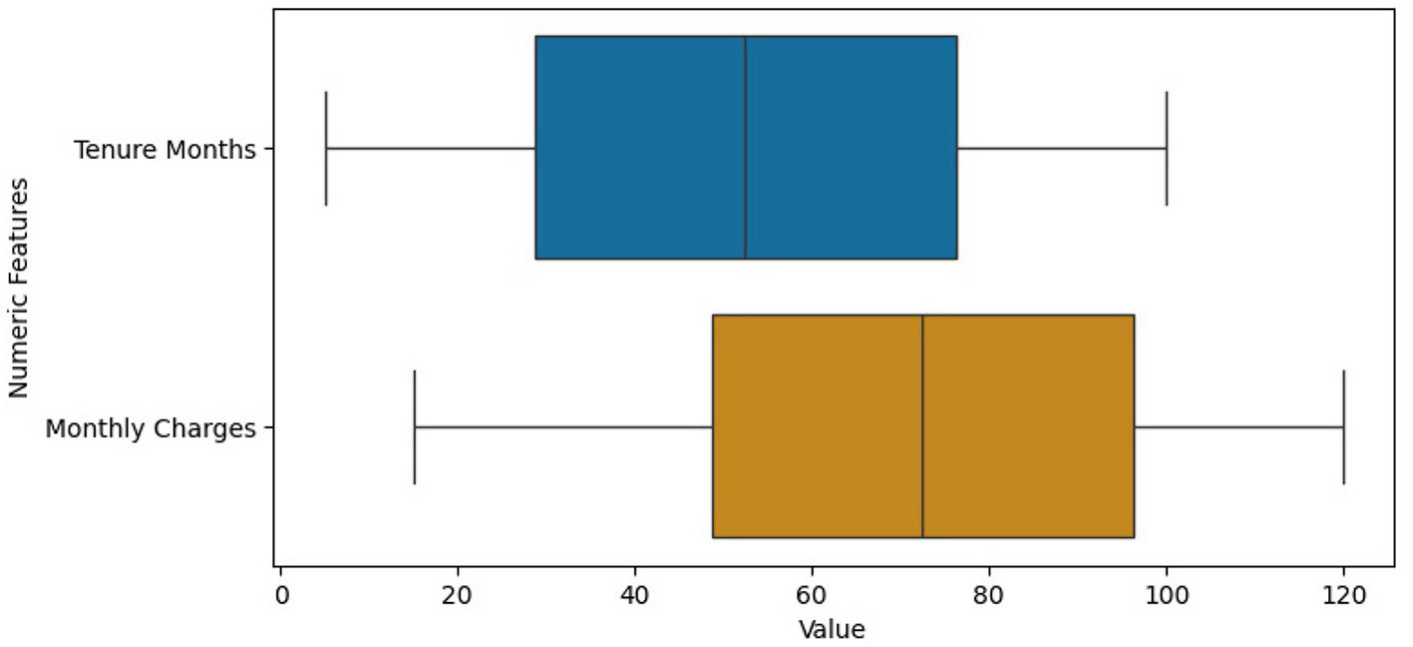
Box-plots of numeric features:“Tenure Months” and “Monthly Charges”.

We used the “.info()” function to get an overview of the structure of the dataset, including its size, the types of data it contains, and whether there are any missing values in the columns. This information is crucial for data cleaning, preprocessing, and initial exploratory data analysis before performing more advanced analyses or building machine learning models with the dataset. Missing values were handled through imputation, using the most frequent value for categorical data and mean for numeric. The histograms in Figure 4 represent the frequency of Tenure Months, Monthly Charges and Total Charges in the dataset. The histogram of Total Charges has symmetrical distribution whereas the histogram of Monthly Charges is slightly left skewed. The box plot in Figure 5 also shows that the dataset has no outliers. It suggests that the data in the specified numerical columns (“Tenure Months” and “Monthly Charges”) do not contain extreme values. This is a good outcome, as it means that the distribution of these numerical features does not deviate significantly from the central tendency. Contract type, monthly charges, and churn value are crucial for the prediction of customer churn. A correlation analysis helps us better understand the correlation between features and target variables.

**Figure 6:**
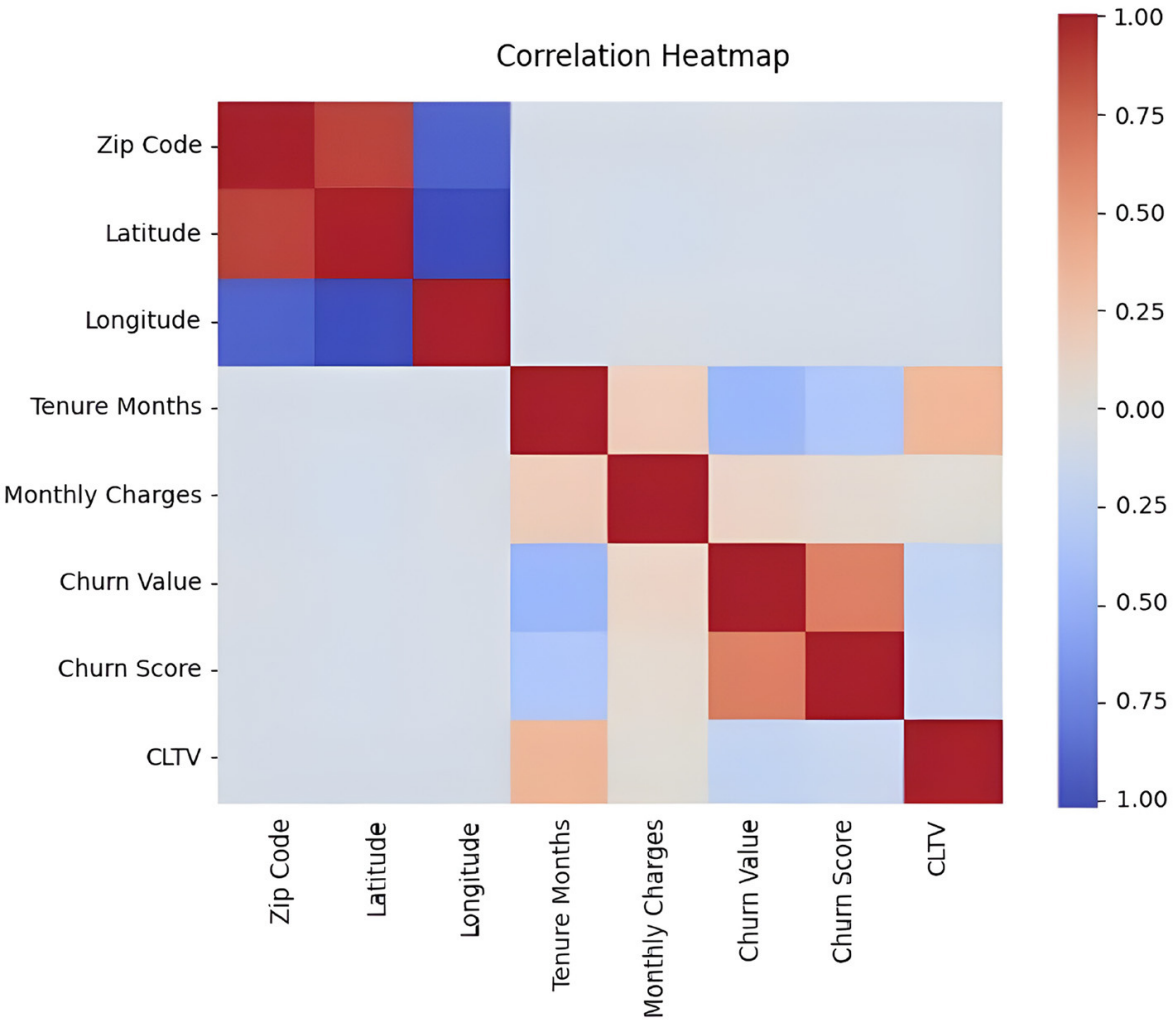
Heatmap of the correlation between different features in the dataset.

As a result, we built a correlation heatmap that shows correlation between different features in the dataset as shown in Figure 6.

### 3.3 Identifying metrics and key performance indicators

Red color represents high correlation and blue color represents low correlation. There is a high correlation between zip code and latitude, CLTV and Tenure Months, Churn value and churn score and low correlation between zip code and longitude, Tenure Months and Churn Value and Tenure Months and Churn score. As a result, based on the EDA conducted, there are two of the features (“Tenure Months” and “Total Charges”) containing empty values in between which had to be converted to *NaN* to proceed. For this, we wrote a code is to ensure that the “Tenure Months” and “Total Charges” columns contain only numeric values and that any non-numeric or missing data is appropriately handled by converting it to NaN). Then, we dropped the rows with missing values (NaN) (By using dropna() with the specified columns, we effectively removed rows with incomplete information in these particular columns, making the data more suitable for analysis and modeling.

In this section, we will identify and analyze metrics and Key Performance Indicators (KPIs) that provide valuable insights into the telecom company's customer churn dynamics. We employ various data exploration and calculation techniques to assess the performance and health of the business. In this study, we identified important business metrics such as churn rate, customer lifetime value (CLTV), average revenue per user (ARPU), customer acquisition cost (CAC), customer retention rate (CRR), and net promoter score (NPS). These KPIs are not just numbers—they help us understand how churn affects the business. For example, a high CAC means it's expensive to get new customers, so keeping current ones is very important. According to the analysis, we found that a churn rate of 26.54 represents the percentage of customers who have churned out of the total number of customers in the data set. This is an important value for businesses to monitor as it reflects customer retention. Furthermore, we calculate the “average lifetime value of the customer” which is the average amount of money the telecom company earns from each customer, which is $4,400.30 in our case. A significant portion of customers, ~ 26.5%, have discontinued their use of telecom services, highlighting a notable churn rate. One key financial metric in the industry is the Average Revenue Per User (ARPU), which represents the average monthly revenue generated by each customer. In this case, the telecom company earns an average of $64.76 per customer per month.

Another crucial factor in business sustainability is the Customer Acquisition Cost (CAC), which reflects the expenses incurred to attract and onboard new customers. For this telecom company, the average cost to acquire a new customer is $8,590.78. Meanwhile, the Customer Retention Rate (CRR) measures the percentage of customers retained over a specific period. In this case, the company has successfully retained 46.92% of its customer base. Finally, the Net Promoter Score (NPS) serves as a key indicator of customer satisfaction and loyalty. It is determined by subtracting the percentage of detractors—customers who rate their experience at 6 or below—from the percentage of promoters, those who score 9 or higher. This metric provides valuable insights into overall customer sentiment and the likelihood of generating positive referrals. We plan to connect the output of our predictive models to these KPIs to show the real impact. For instance, by predicting which high-value customers are likely to leave, the company can take action early and save money. This approach will help telecom companies use our predictions to make better decisions and improve their customer retention strategies.

### 3.4 Cleaning, filtering, and editing data

The data is already standardized (i.e., it has a mean of 0 and a standard deviation of 1), applying the standardization again does not change the values in this case. To impute missing data, on “tenure months” and “Total charges” simple Imputed with strategy = “mean” cannot be used on non-numeric (string) data. This is because the mean strategy is designed for numerical features. In this case, “most frequent” will replace missing categorical values with the most frequent value in each column.

### 3.5 Data transformation and skewness handling

**Figure 7:**
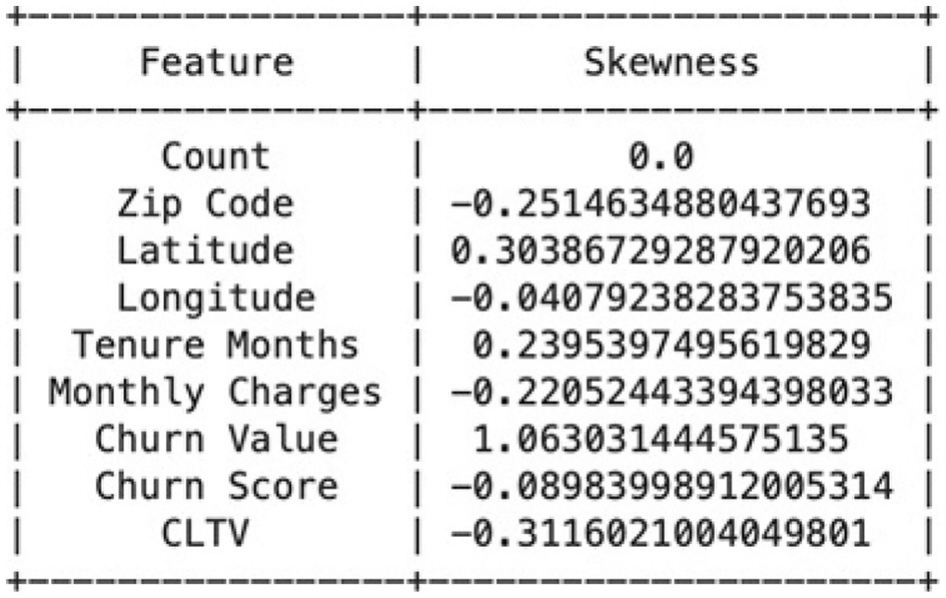
Calculating the skewness of the attributes.

**Figure 8:**
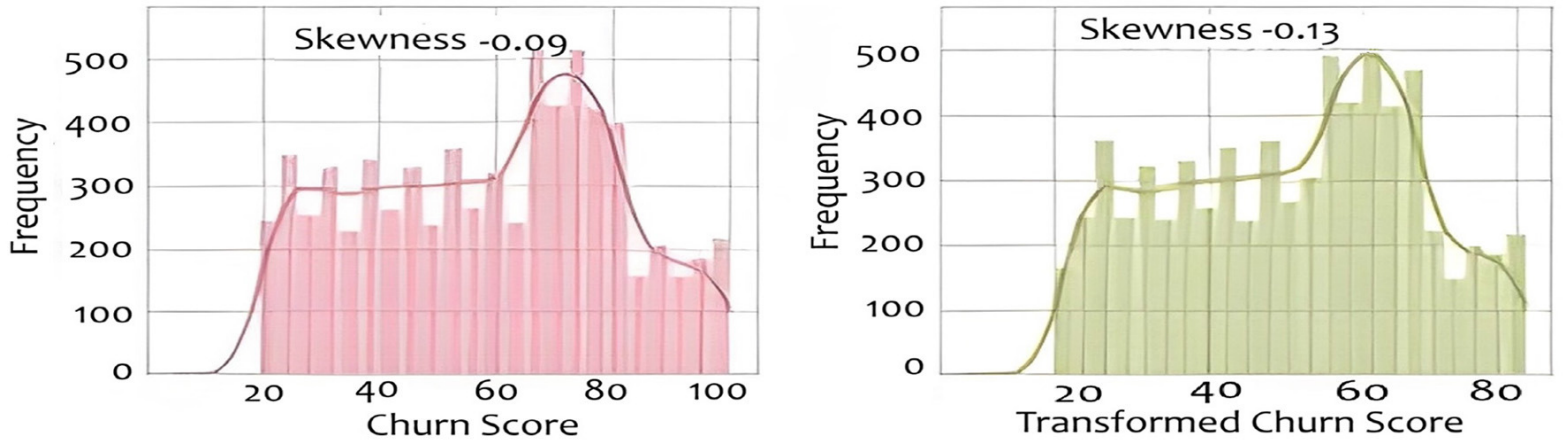
Distribution and transformation of churn scores across the dataset.

Skewed data can impact the performance of machine learning models. We explore various transformation techniques to address skewness in our dataset, ensuring that our features align with the assumptions of predictive modeling. We will also construct various histograms and interpret them to get better understanding. Figure 7 shows the result of Calculating the skewness of the dataset. For example, the count attribute, with a skewness of 0 shows a perfectly symmetrical distribution. The zip Code negative skewness suggests that the distribution is left-skewed. This means that there may be a concentration of values on the right side of the distribution. A positive skewness for Latitude indicates that the distribution is right-skewed. There may be a concentration of values on the left side of the distribution. For the transformation we used 3 different transformation techniques named cbrt, sqrt and reciprocal. We applied that on “Monthly Charges,” “Churn Score” and “Tenure Months.” For example, the “Churn score,” in Figure 8 implies that the distribution of churn scores across the dataset follows a bell- shaped curve characteristic of a normal distribution. The original histogram of the churn score has a skewness of -0.09, indicating a slight leftward skew. After transformations, the best skewness achieved is -0.13. This means that the transformations applied have slightly increased the leftward skewness, but the overall effect is relatively small. For the “Tenure Months,” the histogram is left-skewed and shows frequent growth as the month increases it indicates that a significant portion of customers may have shorter tenures. The skewness before applying transformations was 0.24 and it remained the same even after the transformation techniques. It suggests that the variable may already be approximately normally distributed. In this section, we build and evaluate the performance of four different models which are SVM, Logistic regression and Naïve Bayes. We used Logistic Regression because it is commonly used for binary classification problems, where the target variable has two classes.A linear classifier used as a baseline. It models churn probability through a sigmoid function. It is interpretable and robust with minimal hyperparameter tuning. Our dataset has complex relationships, and we wanted to capture non-linear decision boundaries, so SVM also proved to be a good choice. SVM is well-suited for high-dimensional, non-linear data. We also used KNN because it is a versatile algorithm which considers local patterns. KNN classifies observations based on the majority vote of the k nearest samples. We selected *k* = 5 for cross-validation. Finally, we used Naive Bayes because our data contained categorical features, and we wanted a model with relatively low computational cost. It offers fast training and works well on moderately correlated features. In this study, we applied basic tuning for selected models—such as setting *k* = 5 for KNN based on initial cross-validation tests and choosing the RBF kernel for SVM to handle non-linear data. However, a full hyperparameter optimization process (e.g., Grid Search or Randomized Search) was not conducted across all models. Our focus was to compare model performance under standard and reasonable configurations to establish a strong baseline. We recognize that a more thorough tuning process could further improve model accuracy and generalization, and we plan to include this in future work to enhance model robustness and fairness. The data was split using stratified sampling: 80% for training and 20% for testing to maintain churn distribution. Furthermore, 5-fold cross-validation was used to reduce overfitting and ensure generalization. We applied class weighting in Logistic Regression and SVM to address imbalance (churn rate = 26.5%). We used different evaluation metrics including Accuracy, Precision, Recall (Sensitivity): Correctly predicted churns out of all actual churns, F1-Score and ROC-AUC. Table 1 compares different evaluation methodologies for used models.

**Table 1:** Models performance comparison.

**Model**	**Accuracy**	**Precision**	**Recall**	**F1-Score**	**ROC-AUC**
Logistic regression	89%	0.83	0.75	0.79	0.88
**SVM (RBF)**	**97%**	**0.97**	**0.94**	**0.95**	**0.98**
KNN (*k* = 5)	89%	0.82	0.76	0.78	0.87
Naïve Bayes	88%	0.79	0.73	0.76	0.86

Bold values indicate the highest performance for each metric.

## 4 Predictive models

**Figure 9:**
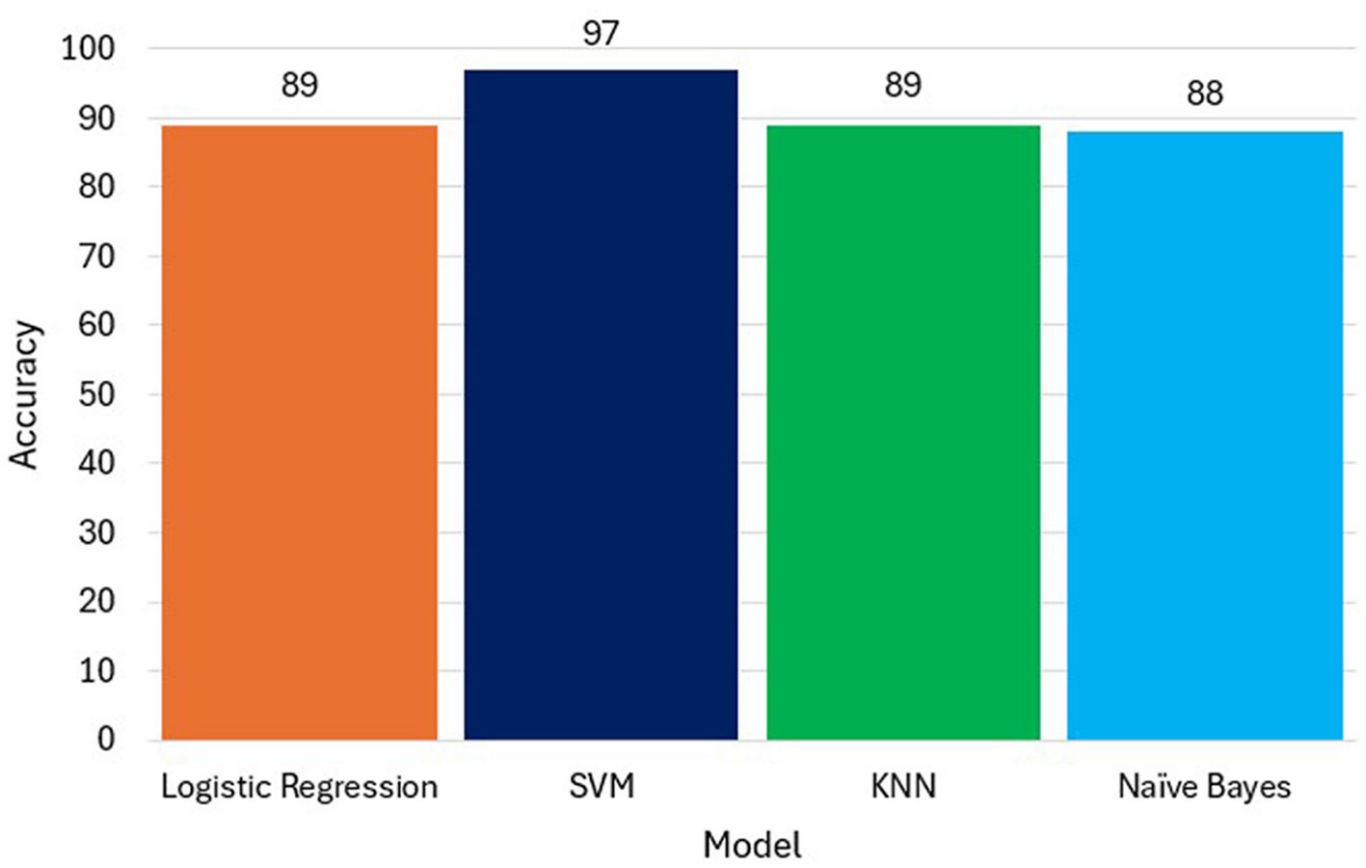
Comparison of models accuracy.

SVM achieved the best results across all metrics, demonstrating strong capability in handling both feature complexity and data imbalance. KNN and Logistic Regression were competitive, with slightly reduced recall. Naïve Bayes, while efficient, was affected by its independence assumptions. This may suggest that the decision boundary created by SVM is effective in separating the classes in the dataset. Whereas, Logistic Regression (89%) and KNN (89%) have similar accuracy scores, but they are lower than SVM. Naive Bayes (88%) has the lowest accuracy among the models see Figure 9 which Compares model accuracy of the built models. This might suggest that the assumption of independence between features might not hold well for our dataset. While our models (Logistic Regression, SVM, KNN, Naive Bayes) provided good accuracy, it's essential to recognize the limitations of predicting future events based on historical data. Our models were good for predicting short-term events but might lack the accuracy to predict long-term future events.

## 5 Conclusion and future work

This study focused on predicting customer churn in the telecommunications sector using advanced data analysis and machine learning techniques. Through data preprocessing, exploratory data analysis, feature selection, and model development, we successfully built predictive models that identify key churn factors. Our analysis highlighted crucial customer attributes such as contract type, monthly charges, and churn value, which significantly influence churn prediction. Among the models tested, Support Vector Machine (SVM) demonstrated the highest accuracy (97%), followed by Logistic Regression (89%), K-Nearest Neighbors (89%), and Naïve Bayes (88%).

While many studies have used the IBM Telco Customer Churn dataset and applied standard models such as SVM, Logistic Regression, KNN, and Naive Bayes, our work stands out in three key ways. First, we focus not only on predictive accuracy but also on aligning the model with business-relevant KPIs such as churn rate, CLTV, ARPU, CAC, CRR, and NPS, offering practical insights for telecom decision-makers. Second, we place strong emphasis on data quality and preprocessing, including a detailed analysis of skewness, outlier detection, and the impact of transformation techniques—areas often underreported in similar works. Third, our framework prioritizes ethical data practices and model transparency, which are frequently overlooked in churn studies. Although we acknowledge the need to enhance interpretability techniques in the current version, this study builds a strong foundation for ethical, practical, and business-aligned churn prediction.

Future research should focus on integrating real-time data streams to enhance churn prediction accuracy and allow telecom companies to take immediate action. Exploring deep learning models, such as recurrent neural networks (RNNs), could further improve predictions by capturing complex patterns in customer behavior. Additionally, incorporating more customer interaction data, such as call logs and sentiment analysis, could refine feature selection and model performance. Lastly, telecom companies can leverage predictive insights to develop personalized retention strategies, offering targeted incentives to high-risk customers and improving overall customer retention.

Furthermore, we plan to apply tools such as SHAP (SHapley Additive exPlanations) and LIME (Local Interpretable Model-Agnostic Explanations) to explain individual predictions and feature contributions in our models. These tools will help telecom decision-makers understand why a customer is predicted to churn, not just whether they will churn. Additionally, we will include feature importance analysis, especially for models like Logistic Regression and Decision Trees, to provide more transparent insights into the factors driving churn.

## Data Availability

Publicly available datasets were analyzed in this study. This data can be found at: https://www.kaggle.com/datasets/yeanzc/telco-customer-churn-ibm-dataset.
